# Attitudes toward Adolescent HPV Vaccination after the COVID-19 Pandemic: A National Survey of Mothers

**DOI:** 10.3390/vaccines12090976

**Published:** 2024-08-28

**Authors:** Gary Glauberman, Erica Liebermann, Melanie L. Kornides, Masako Matsunaga, Eunjung Lim, Gregory Zimet, Holly B. Fontenot

**Affiliations:** 1 School of Nursing, University of Hawaii at Manoa, 2528 McCarthy Mall, Webster Hall, Honolulu, HI 96822, USA; 2 College of Nursing, University of Rhode Island, RINEC 350 Eddy Street, Rm 223, Providence, RI 02903, USA; 3 School of Nursing, University of Pennsylvania, 418 Curie Blvd, Philadelphia, PA 19104, USA; 4 Department of Quantitative Health Sciences, John A. Burns School of Medicine, University of Hawaii at Manoa, Honolulu, HI 96813, USA; 5 Emeritus, Department of Pediatrics, Indiana University School of Medicine, 1625 Sturbridge Road, Indianapolis, IN 46260, USA

**Keywords:** COVID-19, HPV, mothers, pandemic, social media, vaccine hesitancy

## Abstract

In the United States, vaccination rates for many routinely recommended vaccines have recovered to pre-pandemic levels, yet human papillomavirus (HPV) vaccination rates still lag pre-pandemic levels. This study sought to uncover the potential effects of the pandemic on attitudes about the HPV vaccine, and factors associated with changes in attitudes. We conducted a national survey (*n* = 3968) of U.S. mothers with children aged 9–17 years. Outcome variables measured changes in attitude toward the HPV vaccine following the pandemic. Two logistic regression models identified predictors of (1) those who did not have attitude changes (always negative vs. always positive), and (2) those who reported attitude changes (change to negative vs. change to positive). Attitudes toward the HPV vaccine remained unchanged in 78.9% of participants (58.1% positive, 20.8% negative). Of the 21.1% reporting changed attitudes, 9.6% changed to positive and 11.5% to negative. Those reporting changing to a negative attitude had a greater odds of reporting conservative political views, and being unsure/undecided about vaccinating their child against HPV compared to those who reported changing to a positive attitude. Targeted strategies are needed to address erosion in confidence in the HPV vaccine and other vaccines resulting from mis- and disinformation associated with the COVID-19 pandemic and future pandemics.

## 1. Introduction

The COVID-19 pandemic disrupted immunization programs globally, resulting in decreased rates of routinely recommended vaccinations [1,2]. While the rates for many routinely recommended pediatric vaccines in the United States (U.S.) have recovered to pre-pandemic levels, the rate of human papillomavirus (HPV) vaccination still lags pre-pandemic levels [3]. The widespread disruptions caused by the pandemic to adolescent vaccination efforts threaten to slow progress made toward reducing vaccine-preventable diseases, and goals for reducing the incidence of HPV-associated diseases [4].

The Centers for Disease Control and Prevention (CDC) Advisory Committee on Immunization Practices (ACIP) advises that adolescents aged 11–12 years should be vaccinated with tetanus, diphtheria, and acellular pertussis (Tdap), meningococcal conjugate (MenACWY), and HPV vaccines [3]. However, recent reports have highlighted some concerning declines in routine vaccine coverage among adolescents. For example, the latest National Immunization Survey-Teen (2022) noted that adolescents who reached their twelfth birthday during 2020 had lower vaccination coverage, highlighting pandemic disruptions [3]. HPV vaccination initiation, for the first time since 2013, did not increase among adolescents aged 13 to 17 years. The pandemic increased inequity in HPV vaccination initiation, with declining rates compared to pre-pandemic levels, particularly among those insured by Medicaid or uninsured (i.e., those eligible for the Vaccines for Children Program). These were groups that, pre-pandemic, had higher HPV vaccination coverage than those with private insurance [4].

Adolescent vaccine uptake behaviors in the U.S. are influenced by various individual and social factors. Individual parental characteristics (vaccine status, attitudes toward vaccines, risk perception) have been identified as significant influences on parents’ decisions regarding vaccinating their children [5,6,7]. For example, vaccinated parents are more likely than unvaccinated parents to accept vaccines for their children [7]. Additionally, social determinants of health (SDoH), which encompass a wide range of factors in an individual’s social and physical context, also influence individual health behavior decisions, including vaccination. Some of the SDoH that influence child vaccination include parental education, household living conditions and income, health care access, cultural beliefs and religious affiliations, and rural versus urban residency [8]. Furthermore, strong recommendations by health care providers (HCPs) have been linked with higher vaccine acceptance [9,10].

Social media has been shown to positively and negatively affect adolescent vaccination decision-making [11,12]. Increasingly, parents in the U.S. use social media to access health information, including vaccine information [11,12]. While there is growing interest in the role of social media in public health promotion, there are also concerns related to harmful misinformation and disinformation. Negative rhetoric toward vaccines and misinformation distributed via social media crescendoed during the pandemic, and this may have influenced parental attitudes toward obtaining routinely recommended vaccines for their children, resulting in increased vaccine hesitancy among parents [11]. Misinformation spread through social media that politicized the COVID-19 vaccines may have also impacted HPV vaccination rates [13], despite the HPV vaccine being unrelated to the COVID-19 vaccine and the pandemic.

Therefore, this study sought to uncover whether there was a change in mothers’ attitudes toward HPV vaccination due to the pandemic, and factors associated with any change (positive or negative). First, we sought to compare those with unchanged attitudes; then, we compared those who had changes in their attitudes toward the HPV vaccine. We specifically wanted to understand factors related to negative HPV vaccine attitude changes as we believe this will help inform future behavior and attitude change interventions.

## 2. Methods

An online national survey of mothers who live in the U.S. with children aged 9–17 years was conducted in August 2023. We included mothers of children aged 9–17 because HPV vaccination is approved by the U.S. Food and Drug Administration (FDA) for use starting at age 9 [3]. We also focused the study on mothers, who are often the primary parents making health care decisions and interacting with HCPs [14]. Previous research has also noted that women tend to have a higher presence on popular social media platforms (i.e., Instagram, Facebook) than men [15]. Participants were recruited by Dynata™ from their online, large, national, convenience research panel of U.S. residents. Eligibility criteria included being a (1) self-identified female parent or guardian of a child 9 to 17 years old, (2) U.S. resident, and (3) able to read English. Dynata contacted the panel members (who have all agreed to be part of their panel and contacted for research opportunities) whose profiles met the eligibility criteria and provided these potential participants information regarding the survey topic and length, along with a link to participate. Potential participants who clicked on the link to the study’s secure survey software (Qualtrics, Provo, UT, USA) were provided additional information and confirmed their eligibility before providing online consent. All data collected was non-identifiable. With this methodology, the study team does not directly engage in the recruiting process and response rates are not available. Participants were remunerated with a reward point system which is Dynata’s standard practice. The University of Hawaii at Manoa Institutional Review Board approved this study.

## 3. Measures

Socio-demographic and health variables. Socio-demographic characteristics included participants’ age, race/ethnicity, combined annual income, highest level of education, state of primary residence, and whether they lived in an urban, suburban, or rural area. Participants self-reported their political views on a 5-point Likert scale (very liberal to very conservative). Participants also answered if their child had a routine check-up with a HCP in the past 12 months, if their child had received the HPV vaccine (1 or more doses), and if they had a close friend or family member who died or had a serious health consequence from COVID-19. If participants had more than one child aged 9–17 years, they were asked to pick only one of their children for whom they would focus their answers. Lastly, we assessed one item related to COVID-19; how worried are you about the health effects of COVID-19 if your child was infected (assessed on a 5-point Likert scale, 1= not at all worried to 5 = very worried).

Social media variables. Seven social media variables were derived from the survey responses to the questions regarding usage patterns of four popular social media platforms (Instagram, Facebook, TikTok, and Twitter [now known as “X”]) based on preliminary analyses, including exploratory factor analysis. A variable of social media use indicated the number of social media platforms used daily out of the four platforms (range 0 to 4). A variable indicating the degree of influence of each social media platform (Instagram influence, Facebook influence, TikTok influence, and Twitter influence) was created from responses to two questions asking about the perceived credibility and persuasiveness of health messages on the platform. Each variable value is the sum of the two questions with a 6-point Likert scale (range 0 to 10), and a higher value indicates a higher level of positive influence of the platform. The Cronbach’s alpha of the four [social media] influence variables ranged from 0.72 to 0.87, indicating good reliability of the measures. The variable social media negative messages indicated participants’ level of exposure to negative messages about vaccines on social media. The variable was created from responses to four questions for the social media platforms, “How often do you see antivaccine or negative messages about vaccines on [social media]?” The variable value was created by summing the four questions with a 6-point Likert scale, resulting in a value range of 0–20. A higher value indicated a higher exposure to social media negative messages (α = 0.82). A variable of uncertainty about health messages on social media was created from responses to a single question, “Have you seen or heard any information about vaccines on social media that you could not determine were true or false?” with a selection of “No” or “Yes”.

HPV vaccine attitude change. We asked, “Did the COVID-19 pandemic change your attitudes about the HPV vaccine?” Answer choices included the following: (1) Yes, change to more negative attitudes, (2) No, no change, I have always had negative attitudes toward the HPV vaccine, (3) No, no change, I have always had positive attitudes toward the HPV vaccine, and (4) Yes, change to a more positive attitude.

## 4. Statistical Analysis

The characteristics of participants were delineated through the presentation of frequencies and percentages for categorical variables, while mean and standard deviation were calculated for continuous variables. Bivariate analyses examined the relationship between parents’ characteristics and vaccine attitude change or no change during the pandemic. To scrutinize differences across the HPV vaccine attitudes, the chi-squared test was applied for categorical variables, while analysis of variance or the Kruskal–Willis rank sum test was utilized for continuous variables. Lastly, we performed logistic regression analyses to identify factors associated with stable vaccine attitudes and changed vaccine attitudes. Two models were constructed for two comparisons: (1) “having always negative attitudes” vs. “having always positive attitudes (reference)”, i.e., unchanged HPV vaccine attitudes, and (2) “changed to more negative” vs. “changed to more positive”, i.e., changed HPV vaccine attitudes. Variables with a *p*-value less than 0.05 in the univariate logistic regression analyses were included in each multivariable logistic regression model. Unadjusted or adjusted odds ratios (ORs, aORs, respectively) and their 95% confidence intervals (CIs) were obtained from the model results. The goodness of fit was assessed using the Archer–Lemeshow test and the area under the receiver operating characteristics curve, or c-statistics. Sensitivity tests were performed to examine the robustness of the final models. A *p*-value less than 0.05 was considered statistically significant. All analyses were performed using R version 4.2.2 [16].

## 5. Results

Table 1 presents the characteristics of survey participants. In response to the question, “Did the COVID-19 pandemic change your attitude about the HPV vaccine?” more than half indicated “No, no change, I have always had positive attitudes towards HPV vaccine” (58.1%). About one fifth (21.1%) changed to more positive or more negative attitudes (positive: 9.6%; negative 11.5%). The remaining respondents stated, “No, no change, I have always had negative attitudes towards HPV vaccine” (20.8%). The bivariate analysis found that differences in socio-demographic characteristics across the vaccine attitude changes, including the number of children, race/ethnicity, education, neighborhood, child’s health insurance status, whether the child had seen a HCP in the past 12 months, combined annual income, political views, and child’s HPV vaccine status (Table 1). The impact of the pandemic on HPV vaccine attitudes was evident, with perceived severity/experience with COVID-19 and worry about COVID-19 playing significant roles. All scores regarding social media were statistically significant, implying that the use of social media had a discernible effect on attitudes toward HPV vaccines.

Table 2 presents the ORs and 95% CIs from the univariate logistic regression analysis. First, we compared the odds of always having negative attitudes against HPV vaccines to the odds of always having positive attitudes. We found significant associations with this comparison in the number of children aged 9–17, age, race/ethnicity, education, neighborhood, child’s health insurance status, whether the child had seen a HCP in the past 12 months or not, combined annual income, political views, child’s HPV vaccine status, and worry about COVID-19. In the other univariate logistic regression analysis comparing the odds of changing to more negative attitudes with the odds of changing to more positive attitudes, we found more factors were associated with this comparison: number of children aged 9–17, neighborhood, child’s health insurance status, whether the child had seen a HCP in the past 12 months or not, combined annual income, political views, child’s HPV vaccine status, worry about COVID-19, social media use, Instagram influence, Facebook influence, TikTok influence, Twitter influence, and social media negative message.

The above variables were included in a multivariable logistic regression model to identify specific factors associated with the comparisons of always having negative attitudes versus always having positive attitudes. The results, as shown in Table 3, indicate that non-Hispanic (NH) Black participants had significantly higher odds of always having a negative attitude (aOR = 2.51, 95% CI = 1.81–3.45) compared to NH White participants. Educational attainment, political views, and a child’s HPV vaccine status were also significantly associated with the likelihood of always having negative attitudes. Participants with some college or associate degree had a lower odds of always having a negative attitude (aOR = 0.49, 95% CI = 0.38–0.63) when compared to those with a high school degree, GED, or less. Similarly, those with a bachelor’s degree (aOR = 0.51, 95% CI = 0.37–0.70) or a graduate degree (aOR = 0.35, 95% CI = 0.23–0.52) had even lower odds of always having a negative attitude. On the other hand, participants who identified as “conservative” (aOR = 1.56, 95% CI = 1.14–2.14), “moderate/middle of the road” (aOR = 1.40, 95% CI = 1.05–1.87), or “chose not to answer” their political views (aOR = 1.54, 95% CI = 1.05–2.27) had a higher odds of always having a negative attitude. Participants who expressed no intention of getting their child the HPV vaccine or who were unsure/undecided about the vaccine had a significantly higher odds of always having a negative attitude. Specifically, those who did not plan to vaccinate their child had extremely high odds of a negative attitude (aOR = 35.02, 95% CI = 26.36–47.00), while those who were unsure/undecided also had increased odds (aOR = 5.36, 95% CI = 4.24–6.78). However, an increase in worry about COVID-19 was associated with a decrease in the odds of always having a negative attitude (aOR = 0.91, 95% CI = 0.83–0.99).

The results for the second comparison, which examined changing to more negative attitudes vs. changing to more positive attitudes toward the HPV vaccine (Table 3), indicate that NH Asian participants had a lower odds of reporting an attitude change to the negative (aOR = 0.37, 95% CI = 0.13–0.97) compared to NH White participants. Those living in an urban neighborhood were less likely to change to a negative attitude compared to those in a rural neighborhood (aOR = 0.59, 95% CI = 0.35–0.98). On the other hand, those with an annual income of USD 50,000–99,999 had a higher odds of a change to a negative attitude compared to those with an income of USD 49,999 or less (aOR = 2.00, 95% CI = 1.93–3.13). Participants with political views other than liberal were also more likely to change to a negative attitude: conservatives (aOR = 1.93, 95% CI = 1.16–3.23), those with moderate/middle of the road views (aOR = 1.98, 95% CI = 1.22–3.24), and those who preferred not to disclose their political views (aOR = 3.74, 95% CI = 1.67–8.67). Parents who expressed no intention of vaccinating their child against HPV or were unsure/undecided had significantly higher odds of changing to a negative attitude, especially those with no intention of vaccinating (aOR = 19.42, 95% CI = 10.78–37.52) and those who were unsure/undecided (aOR = 5.42, 95% CI = 3.59–8.30). In terms of other factors, worry about COVID-19 and social media variables were not associated with a change to a negative attitude, except for TikTok influence: increased influence from TikTok was associated with a decreased odds of a change to a negative attitude toward HPV vaccination (aOR = 0.91, 95% CI = 0.83–0.99).

The variable selection criterion for multivariable regression analysis resulted in the model including race/ethnicity but not the U.S. regions in both comparisons. Considering that the race/ethnicity distributions vary across the regions, we performed sensitivity tests by adding the U.S. region to each comparison model. The sensitivity test results were similar, although the test for the first comparison additionally found that those in East North/South Central and Mountain regions were less likely to be always negative compared to those in the Pacific region (*p* < 0.05).

## 6. Discussion

Among this national sample of mothers of children aged 9–17 years, attitudes toward the HPV vaccine remained positive and unchanged in 78.9% of the participants. However, 21.1% of participants reported changing attitudes about the HPV vaccine after the pandemic. Specifically, 58.1% of participants reported having consistently positive attitudes toward the HPV vaccine, whereas 20.8% reported consistently negative attitudes. Of those who had a change in attitude, a larger proportion reported attitudes changing to more negative (11.5%) when compared to the proportion changing to more positive (9.6%). These results highlight that approximately 20% of HPV vaccine attitudes were variable and malleable; therefore, targeted interventions focused on reducing vaccine hesitancy among those who had negative attitude changes are necessary. Negative parental attitudes toward HPV vaccines are known to contribute to vaccine hesitancy [17]. This study suggests that negative attitudinal changes toward HPV vaccination during the pandemic may partially explain the lack of rebound in HPV vaccine uptake as compared to other routinely recommended vaccines in the immediate years following the pandemic [3]. Furthermore, the findings suggest that we should anticipate that future pandemics may further erode confidence in the HPV vaccine and other vaccines, indicating that efforts at pandemic preparedness should include interventions emphasizing the importance of vaccination as an essential approach to health maintenance.

This study identified factors associated with stable HPV vaccine attitudes as well as changes in HPV vaccine attitudes. Participants who identified as NH Black, had any other political views other than liberal, and had no intention to or were unsure/undecided about obtaining the HPV vaccine for their child were most likely to always have negative attitudes toward HPV vaccines. Political views other than liberal and lack of intention or indecision about obtaining the HPV vaccine were also predictors of a change to a negative attitude. These political influences are consistent with other recent reports on political views and vaccine attitudes [18,19]. There appears to be a political divide that is related to vaccine attitudes, a trend that became apparent during the COVID-19 pandemic, with persons identifying as more conservative generally having more negative attitudes toward vaccines as compared to persons who identify with more liberal political views [20,21]. Those planning future vaccine interventions may find it beneficial to identify and engage with conservative community leaders and influencers to counteract the negative trend in attitudes about vaccination. In contrast, participants who had higher levels of education and increased worry about COVID-19 were most likely to always have positive attitudes toward HPV vaccines. Plus, those who identified as NH Asian, lived in urban areas, and reported increased influence from TikTok were predictors of a change to a positive attitude toward the HPV vaccine after the pandemic.

Additionally, social media has become a major source of information about vaccines. While these social media platforms are effective tools for rapidly sharing information and communicating widely to broad audiences, they have also amplified the dissemination of misinformation, exacerbating vaccine hesitancy [22,23,24]. Middleman et al. found that the proportion of parents and adolescents who reported that what they read on social media about vaccine safety had concerned them had increased over ten months during the pandemic [25]. In our study, social media variables were not associated with negative attitudes nor changing to negative attitudes toward HPV vaccines. Interestingly, increased TikTok influence was found to decrease the odds of changing to a negative attitude, which suggests that the impact of social media may be dependent on the beliefs and viewpoints of the persons or organizations that individuals follow on social media. A previous systematic review reported that social media can improve HPV vaccine awareness, and can have either negative or positive effects on attitudes toward the HPV vaccine depending on the content of social media posts and the presence of anti-vaccine messaging [26]. A few studies have examined how social media interventions can be leveraged to improve vaccine attitudes and uptake [27,28]. Grosso et al. found that social media messaging regarding COVID-19 vaccines had a positive impact on COVID-19 vaccination rates among children [27]. Buller et al. also reported an increase in HPV vaccination rates as a result of a Facebook intervention with mothers of adolescent girls [28].

## 7. Implications

It is not known whether the net negative change in attitudes toward the HPV vaccine due to the pandemic will continue in this direction. However, to regain lost ground on HPV vaccine hesitancy in the years following the pandemic, renewed efforts are needed by clinicians and public health professionals to counteract this trend. For one, HCPs play an important role in reducing HPV vaccine hesitancy. If parents are hesitant to vaccinate their children, providers should explore changes in attitudes that may have occurred since the pandemic and be ready to address vaccine hesitancy. Prior studies have demonstrated the importance of HCPs’ recommendations in increasing HPV vaccine uptake among adolescents [29,30], and that the HCP’s engagement of parents through eliciting questions and providing information and support can decrease vaccine hesitancy and improve uptake [31,32]. For example, there is some evidence that the use of motivational interviewing techniques can be an effective way to engage with some vaccine-hesitant parents and to address their concerns [33].

A greater understanding of how social media might be utilized to improve vaccine attitudes and reduce vaccine hesitancy is needed. This study indicated the need to invest more effort into leveraging the influence of social media tools (i.e., TikTok) as a channel for transmitting accurate, trustworthy health communication. A recently published qualitative study among parents highlighted fatigue from contentious vaccine-related content on social media and a desire for vaccine messaging on social media to be from recognizable health institutions with links to reputable resources [34]. To this end, HCPs in practice and public health campaigns must employ best practices for counterbalancing negative messages on social media by providing factual information and including links to refer viewers to where to learn more.

## 8. Limitations and Strengths

This study was limited by its cross-sectional design, precluding an ability to determine the causality of associations found with changes in HPV vaccine attitudes. Only female-identifying parents/caregivers were invited to participate in the survey, and while these individuals more often serve as the primary decision-makers for their child’s health, the results may have differed if male-identifying parents were included. Political views were self-assessed, while actual political affiliation was not assessed. Data was not collected regarding the timing of HPV vaccination for the one child for which they focused their answers, just if they had or had not vaccinated that child. The variable assessing participants’ worry about how COVID-19 would affect their child may have differed if it assessed worry regarding how COVID-19 would affect the adult participants (or the adult’s loved ones). A strength of the study was the inclusion of a large, diverse U.S. national sample of mothers of children aged 9–17 years. The large national sample was facilitated by our recruitment methodology; however, future research may include diverse methods for recruitment, including in-person recruitment. Future longitudinal research is needed to determine whether the pandemic has had a lasting influence on HPV vaccine attitudes, and whether past HPV vaccine intervention strategies remain effective or if interventions need to be modified to account for new factors that influence vaccine attitudes after the pandemic.

## 9. Conclusions

This study found that the COVID-19 pandemic coincided with and may have contributed to a small “net-negative” shift in attitudes toward the HPV vaccine, despite HPV vaccination not being directly related to the pandemic. Opportunities to make up ground lost during the pandemic include developing public health messaging tailored to communities in which there may be higher levels of HPV vaccine hesitancy, increasing HCP involvement in addressing HPV vaccine hesitancy among parents, and leveraging the influence of new social media tools as channels for transmitting accurate, trustworthy information about vaccines.

## Figures and Tables

**Table 1:** Characteristics of female participants who have child(ren) aged 9–17 years old: U.S. National Online Survey 2023.

	Attitudes toward HPV Vaccination					
	Overall *n* = 3968	Always Positive *n* = 2304 (58.1%)	Changed to More Positive *n* = 379 (9.6%)	Changed to more Negative *n* = 458 (11.5%)	Always Negative *n* = 827 (20.8%)	*p* ^8^
Number of children aged 9–17, *n* (%)						**0.038**
1 child	2171 (54.7)	1282 (55.6)	224 (59.1)	232 (50.7)	433 (52.4)	
2 children	1331 (33.5)	771 (33.5)	119 (31.4)	163 (35.6)	278 (33.6)	
3 or more children	466 (11.7)	251 (10.9)	36 (9.5)	63 (13.8)	116 (14.0)	
Age, *n* (%)						0.073
34 years or younger	749 (18.9)	416 (18.1)	76 (20.1)	87 (19.0)	170 (20.6)	
35–44 years	1937 (48.8)	1129 (49)	174 (45.9)	224 (48.9)	410 (49.6)	
45–54 years	1010 (25.5)	591 (25.7)	91 (24.0)	121 (26.4)	207 (25.0)	
55 years or older	272 (6.9)	168 (7.3)	38 (10.0)	26 (5.7)	40 (4.8)	
Race/Ethnicity, *n* (%)						**<0.001**
NH White	2934 (73.9)	1787 (77.6)	221 (58.3)	328 (71.6)	598 (72.3)	
Hispanic	356 (9.0)	191 (8.3)	55 (14.5)	49 (10.7)	61 (7.4)	
NH Black	431 (10.9)	196 (8.5)	69 (18.2)	51 (11.1)	115 (13.9)	
NH Asian	113 (2.8)	67 (2.9)	19 (5.0)	10 (2.2)	17 (2.1)	
Native American	58 (1.5)	29 (1.3)	5 (1.3)	9 (2.0)	15 (1.8)	
Mixed Race	76 (1.9)	34 (1.5)	10 (2.6)	11 (2.4)	21 (2.5)	
Education, *n* (%)						**<0.001**
High school, GED, or less	937 (23.6)	461 (20)	102 (26.9)	103 (22.5)	271 (32.8)	
Some college or associate degree	1590 (40.1)	958 (41.6)	122 (32.2)	180 (39.3)	330 (39.9)	
Bachelor’s degree	932 (23.5)	526 (22.8)	119 (31.4)	128 (27.9)	159 (19.2)	
Graduate degree	509 (12.8)	359 (15.6)	36 (9.5)	47 (10.3)	67 (8.1)	
US Region, *n* (%)						0.534
Pacific	386 (9.7)	209 (9.1)	45 (11.9)	46 (10.0)	86 (10.4)	
East North Central	624 (15.7)	371 (16.1)	52 (13.7)	70 (15.3)	131 (15.8)	
East South Central	333 (8.4)	197 (8.6)	31 (8.2)	41 (9.0)	64 (7.7)	
Middle Atlantic	480 (12.1)	282 (12.2)	44 (11.6)	48 (10.5)	106 (12.8)	
Mountain	267 (6.7)	157 (6.8)	24 (6.3)	33 (7.2)	53 (6.4)	
New England	153 (3.9)	98 (4.3)	17 (4.5)	16 (3.5)	22 (2.7)	
South Atlantic	892 (22.5)	504 (21.9)	91 (24.0)	105 (22.9)	192 (23.2)	
West North Central	294 (7.4)	192 (8.3)	25 (6.6)	29 (6.3)	48 (5.8)	
West South Central	539 (13.6)	294 (12.8)	50 (13.2)	70 (15.3)	125 (15.1)	
Neighborhood, *n* (%)						**<0.001**
Rural	1045 (26.3)	587 (25.5)	82 (21.6)	122 (26.6)	254 (30.7)	
Suburban	2059 (51.9)	1262 (54.8)	166 (43.8)	255 (55.7)	376 (45.5)	
Urban	864 (21.8)	455 (19.7)	131 (34.6)	81 (17.7)	197 (23.8)	
Child’s health insurance status ^1^, *n* (%)						**<0.001**
Private insurance	2052 (51.7)	1256 (54.5)	166 (43.8)	248 (54.1)	382 (46.2)	
Public insurance	1779 (44.8)	978 (42.4)	200 (52.8)	192 (41.9)	409 (49.5)	
Not sure/ do not know	137 (3.5)	70 (3)	13 (3.4)	18 (3.9)	36 (4.4)	
Child Seen HCP in the past 12 mon, *n* (%)						**<0.001**
No	304 (7.7)	146 (6.3)	20 (5.3)	46 (10.0)	92 (11.1)	
Yes	3664 (92.3)	2158 (93.7)	359 (94.7)	412 (90.0)	735 (88.9)	
Combined annual income, *n* (%)						**<0.001**
USD 49,999 or less	1426 (35.9)	779 (33.8)	170 (44.9)	149 (32.5)	328 (39.7)	
USD 50,000 to 99,999	1453 (36.6)	831 (36.1)	122 (32.2)	185 (40.4)	315 (38.1)	
USD 100,000 or more	985 (24.8)	640 (27.8)	77 (20.3)	112 (24.5)	156 (18.9)	
Choose not to answer	104 (2.6)	54 (2.3)	10 (2.6)	12 (2.6)	28 (3.4)	
Political views, *n* (%)						**<0.001**
Liberal	878 (22.1)	605 (26.3)	111 (29.3)	55 (12.0)	107 (12.9)	
Conservative	1106 (27.9)	548 (23.8)	109 (28.8)	185 (40.4)	264 (31.9)	
Moderate/middle of the road	1609 (40.5)	949 (41.2)	142 (37.5)	173 (37.8)	345 (41.7)	
Choose not to answer	375 (9.5)	202 (8.8)	17 (4.5)	45 (9.8)	111 (13.4)	
Child’s HPV Vaccine Status, ^2^ *n* (%)						**<0.001**
Yes	2604 (65.6)	1897 (82.3)	312 (82.3)	150 (32.8)	245 (29.6)	
No, I do not plan	640 (16.1)	88 (3.8)	14 (3.7)	163 (35.6)	375 (45.3)	
Unsure or undecided	724 (18.2)	319 (13.8)	53 (14.0)	145 (31.7)	207 (25.0)	
Perceived severity/experience with COVID-19, *n* (%)						**0.037**
No	2432 (61.3)	1423 (61.8)	207 (54.6)	280 (61.1)	522 (63.1)	
Yes	1536 (38.7)	881 (38.2)	172 (45.4)	178 (38.9)	305 (36.9)	
Worry COVID-19, ^3^ mean (SD)	2.5 (1.3)	2.6 (1.2)	3.0 (1.3)	2.3 (1.3)	2.2 (1.2)	**<0.001**
Social media use, ^4^ mean (SD)	1.6 (1.0)	1.6 (1)	1.9 (1.2)	1.6 (1.0)	1.5 (1.0)	**<0.001**
Instagram influence, ^5^ mean (SD)	3.6 (2.5)	3.5 (2.4)	5.2 (2.9)	3.5 (2.5)	3.5 (2.5)	**<0.001**
Facebook influence, ^5^ mean (SD)	4.3 (2.2)	4.2 (2.1)	5.6 (2.6)	4.2 (2.1)	4.2 (2.2)	**<0.001**
TikTok influence, ^5^ mean (SD)	3.3 (2.7)	3.1 (2.5)	4.8 (3.1)	3.1 (2.7)	3.2 (2.8)	**<0.001**
Twitter influence, ^5^ mean (SD)	2.3 (2.7)	2.2 (2.5)	3.9 (3.3)	2.3 (2.6)	2.2 (2.7)	**<0.001**
Social media negative messages, ^6^						
mean (SD)	5.3 (4.7)	4.8 (4.3)	7.8 (6.1)	6.0 (5.1)	5.1 (4.6)	**<0.001**
Uncertainty of social media messages, ^7^ *n* (%)						**<0.001**
No	2196 (55.3)	1329 (57.7)	192 (50.7)	213 (46.5)	462 (55.9)	
Yes	1772 (44.7)	975 (42.3)	187 (49.3)	245 (53.5)	365 (44.1)	

HCP: health care provider. HPV: human papillomavirus. NH: Non-Hispanic. SD: standard deviation. ^1^ Private insurance includes HMO, PPO, and others. Public insurance includes Medicaid, Medicare, other state programs, and TRICARE/Military. ^2^ Responses to the question “Has [child] received the HPV vaccine?” Yes included “Yes, they are vaccinated” and “No, but I plan to get them vaccinated”. ^3^ Likert scale for worry about COVID-19 (potential range: 1–5). ^4^ The number of social media platforms used daily (potential range: 0–4). ^5^ Total score on the Likert scale for credibility and persuasiveness of the social media platform (potential range: 0–10). ^6^ Total score on the Likert scale for the frequency of exposure to negative messages on each social media platform (potential range: 0–20). ^7^ Responses to the question “Have you seen or heard any information about vaccines on social media that you could not determine were true or false?”. ^8^ *p*-values less than 0.05 are in bold text.

**Table 2:** Odds ratios and 95% confidence intervals of socio-demographic characteristics and social media variables: univariate logistic regression analysis of attitude against the HPV vaccine: U.S. National Online Survey 2023.

	Attitudes toward HPV Vaccination			
	Always Negative vs. Always Positive (Ref.) (*n* = 3131)		Changed to More Negative vs. Changed to More Positive (Ref.) (*n* = 837)	
	OR (95% CI) ^8^	*p* ^9^	OR (95% CI) ^8^	*p* ^9^
Number of children aged 9–17				
1 child	Reference	**0.047**	Reference	**0.030**
2 children	1.07 (0.90, 1.27)		1.32 (0.98, 1.79)	
3 or more children	**1.37 (1.07, 1.75)**		**1.69 (1.08, 2.67)**	
Age				
34 years or younger	Reference	**0.045**	Reference	0.107
35–44 years	0.89 (0.72, 1.10)		1.12 (0.78, 1.62)	
45–54 years	0.86 (0.68, 1.09)		1.16 (0.77, 1.75)	
55 years or older	**0.58 (0.39, 0.85)**		0.60 (0.33, 1.07)	
Race/Ethnicity				
NH White	Reference	**<0.001**	Reference	**<0.001**
Hispanic	0.95 (0.70, 1.28)		**0.60 (0.39, 0.91)**	
NH Black	**1.75 (1.37, 2.24)**		**0.50 (0.33, 0.74)**	
NH Asian	0.76 (0.43, 1.27)		**0.35 (0.16, 0.76)**	
Native American	1.55 (0.80, 2.86)		1.21 (0.41, 3.99)	
Mixed Race	**1.85 (1.05, 3.18)**		0.74 (0.31, 1.81)	
Education				
High school, GED, or less	Reference	**<0.001**	Reference	0.136
Some college or associate degree	**0.59 (0.48, 0.71)**		**1.46 (1.02, 2.09)**	
Bachelor’s degree	**0.51 (0.41, 0.65)**		1.07 (0.74, 1.54)	
Graduate degree	**0.32 (0.23, 0.43)**		1.29 (0.78, 2.17)	
US Region				
Pacific	Reference	0.067	Reference	0.942
East North Central	0.86 (0.62, 1.18)		1.32 (0.76, 2.28)	
East South Central	0.79 (0.54, 1.15)		1.29 (0.70, 2.42)	
Middle Atlantic	0.91 (0.65, 1.28)		1.07 (0.60, 1.91)	
Mountain	0.82 (0.55, 1.22)		1.35 (0.69, 2.64)	
New England	**0.55 (0.32, 0.91)**		0.92 (0.41, 2.05)	
South Atlantic	0.93 (0.69, 1.25)		1.13 (0.69, 1.86)	
West North Central	**0.61 (0.40, 0.91)**		1.13 (0.58, 2.24)	
West South Central	1.03 (0.75, 1.44)		1.37 (0.79, 2.38)	
Neighborhood				
Rural	Reference	**<0.001**	Reference	**<0.001**
Suburban	**0.69 (0.57, 0.83)**		1.03 (0.73, 1.45)	
Urban	1.00 (0.80, 1.25)		**0.42 (0.28, 0.61)**	
Child’s health insurance status ^1^				
Private insurance	Reference	**<0.001**	Reference	**0.007**
Public insurance	**1.38 (1.17, 1.62)**		**0.64 (0.49, 0.85)**	
Not sure/ do not know	**1.69 (1.10, 2.55)**		0.93 (0.44, 1.98)	
Child Seen HCP in the past 12 mon		**<0.001**		**0.010**
No	Reference		Reference	
Yes	**0.54 (0.41, 0.71)**		**0.50 (0.28, 0.85)**	
Combined annual income				
USD 49,999 or less	Reference	**<0.001**	Reference	**0.003**
USD 50,000 to 99,999	0.90 (0.75, 1.08)		**1.73 (1.26, 2.38)**	
USD 100,000 or more	**0.58 (0.46, 0.72)**		**1.66 (1.15, 2.39)**	
Choose not to answer	1.23 (0.76, 1.96)		1.37 (0.57, 3.33)	
Political views				
Liberal	Reference	**<0.001**	Reference	**<0.001**
Conservative	**2.72 (2.12, 3.52)**		**3.43 (2.30, 5.14)**	
Moderate/middle of the road	**2.06 (1.62, 2.62)**		**2.46 (1.67, 3.66)**	
Choose not to answer	**3.11 (2.28, 4.24)**		**5.34 (2.85, 10.41)**	
Child’s HPV Vaccine Status ^2^				
Yes	Reference	**<0.001**	Reference	**<0.001**
No, I do not plan	**33.00 (25.36, 43.33)**		**24.22 (14.03, 45.08)**	
Unsure or undecided	**5.02 (4.03, 6.26)**		**5.69 (3.95, 8.29)**	
Perceived severity/experience with COVID-19				
No	Reference	0.489	Reference	0.057
Yes	0.94 (0.80, 1.11)		0.77 (0.58, 1.01)	
Worry COVID-19 ^3^	**0.78 (0.73, 0.84)**	**<0.001**	**0.69 (0.62, 0.77)**	**<0.001**
Social media use ^4^	0.98 (0.90, 1.06)	0.587	**0.74 (0.66, 0.84)**	**<0.001**
Instagram influence ^5^	1.01 (0.97, 1.04)	0.750	**0.80 (0.76, 0.85)**	**<0.001**
Facebook influence ^5^	1.01 (0.97, 1.05)	0.682	**0.77 (0.72, 0.82)**	**<0.001**
TikTok influence ^5^	1.02 (0.99, 1.05)	0.153	**0.81 (0.77, 0.85)**	**<0.001**
Twitter influence ^5^	1.01 (0.97, 1.04)	0.742	**0.83 (0.80, 0.87)**	**<0.001**
Social media negative messages ^6^	1.01 (0.99, 1.03)	0.159	**0.94 (0.92, 0.97)**	**<0.001**
Uncertainty of social media messages ^7^				
No	Reference	0.365	Reference	0.231
Yes	1.08 (0.92, 1.26)		1.18 (0.90, 1.55)	

OR: odds ratio. CI: confidence interval. HCP: health care provider. HPV: human papillomavirus. NH: Non-Hispanic. Ref.: Reference. ^1^ Private insurance includes HMO, PPO, and others. Public insurance includes Medicaid, Medicare, other state programs, and TRICARE/Military. ^2^ Responses to the question “Has [child] received the HPV vaccine?” Yes included “Yes, they are vaccinated” and “No, but I plan to get them vaccinated”. ^3^ Likert scale for worry about COVID-19 (potential range: 1–5). ^4^ The number of social media platforms used daily (potential range: 0–4). ^5^ Total score on the Likert scale for credibility and persuasiveness of the social media platform (potential range: 0–10). ^6^ Total score on the Likert scale for the frequency of exposure to negative messages on each social media platform (potential range: 0–20). ^7^ Responses to the question “Have you seen or heard any information about vaccines on social media that you could not determine were true or false?”. ^8^ Bold fonts indicate a local *p*-value less than 0.05. ^9^ Bold fonts indicate a global *p*-value less than 0.05.

**Table 3:** Adjusted odds ratios and 95% confidence intervals of socio-demographic characteristics and social media variables: multivariate logistic regression analysis of attitude against the HPV vaccine: U.S. National Online Survey 2023.

	Attitudes toward HPV Vaccination	
	Always Negative vs. Always Positive (Ref.) ^7^ (*n* = 3131)	Changed to More Negative vs. Changed to More Positive (Ref.) ^9^ (*n* = 837)
	aOR (95% CI) ^8^	aOR (95% CI) ^8^
Number of children aged 9–17		
1 child	Reference	Reference
2 children	1.13 (0.90, 1.41)	1.19 (0.81, 1.76)
3 or more children	1.33 (0.97, 1.82)	1.28 (0.73, 2.26)
Age		
34 years or younger	Reference	
35–44 years	0.95 (0.73, 1.25)	
45–54 years	1.31 (0.96, 1.78)	
55 years or older	0.88 (0.54, 1.40)	
Race/Ethnicity		
NH White	Reference	Reference
Hispanic	1.24 (0.85, 1.78)	0.94 (0.55, 1.63)
NH Black	**2.51 (1.81, 3.45)**	0.83 (0.48, 1.41)
NH Asian	1.00 (0.51, 1.89)	**0.37 (0.13, 0.97)**
Native American	1.51 (0.69, 3.19)	0.92 (0.24, 3.93)
Mixed Race	1.39 (0.63, 2.93)	0.87 (0.29, 2.63)
Education		
High school, GED, or less	Reference	
Some college or associate degree	**0.49 (0.38, 0.63)**	
Bachelor’s degree	**0.51 (0.37, 0.70)**	
Graduate degree	**0.35 (0.23, 0.52)**	
Neighborhood		
Rural	Reference	Reference
Suburban	0.90 (0.70, 1.15)	0.98 (0.62, 1.55)
Urban	1.31 (0.98, 1.76)	**0.59 (0.35, 0.98)**
Child’s health insurance status ^1^		
Private insurance	Reference	Reference
Public insurance	1.15 (0.90, 1.47)	0.72 (0.48, 1.07)
Not sure/ do not know	1.02 (0.58, 1.77)	0.71 (0.26, 1.95)
Child Seen HCP in the past 12 mon		
No	Reference	Reference
Yes	1.13 (0.79, 1.63)	0.79 (0.39, 1.55)
Combined annual income		
USD 49,999 or less	Reference	Reference
USD 50,000 to 99,999	1.07 (0.83, 1.38)	**2.00 (1.29, 3.13)**
USD 100,000 or more	0.84 (0.60, 1.17)	1.64 (0.95, 2.85)
Choose not to answer	1.04 (0.56, 1.90)	1.17 (0.36, 3.68)
Political views		
Liberal	Reference	Reference
Conservative	**1.56 (1.14, 2.14)**	**1.93 (1.16, 3.23)**
Moderate/middle of the road	**1.40 (1.05, 1.87)**	**1.98 (1.22, 3.24)**
Choose not to answer	**1.54 (1.05, 2.27)**	**3.74 (1.67, 8.67)**
Child’s HPV Vaccine Status ^2^		
Yes	Reference	Reference
No, I do not plan	**35.02 (26.36, 47.00)**	**19.42 (10.78, 37.52)**
Unsure or undecided	**5.36 (4.24, 6.78)**	**5.42 (3.59, 8.30)**
Worry COVID-19 ^3^	**0.91 (0.83, 0.99)**	0.92 (0.80, 1.06)
Social media use ^4^		0.99 (0.82, 1.18)
Instagram influence ^5^		0.94 (0.86, 1.03)
Facebook influence ^5^		0.93 (0.84, 1.03)
TikTok influence ^5^		**0.91 (0.83, 0.99)**
Twitter influence ^5^		0.97 (0.89, 1.04)
Social media negative messages ^6^		1.01 (0.97, 1.05)

aOR: adjusted odds ratio. CI: confidence interval. HCP: health care provider. HPV: human papillomavirus. NH: Non-Hispanic. Ref.: Reference. ^1^ Private insurance includes HMO, PPO, and others. Public insurance includes Medicaid, Medicare, other state programs, and TRICARE/Military. ^2^ Responses to the question “Has [child] received the HPV vaccine?” Yes included “Yes, they are vaccinated” and “No, but I plan to get them vaccinated”. ^3^ Likert scale for worry about COVID-19 (potential range: 1–5). ^4^ The number of social media platforms used daily (potential range: 0–4). ^5^ Total score on the Likert scale for credibility and persuasiveness of the social media platform (potential range: 0–10). ^6^ Total score on the Likert scale for the frequency of exposure to negative messages on each social media platform (potential range: 0–20). ^7^ The model fit was acceptable (goodness of fit, *p* = 0.40; area under the curve 0.74). ^8^ Bold fonts indicate a local *p*-value less than 0.05. ^9^ The model fit was acceptable (goodness of fit, *p* = 0.97; area under the curve 0.79).

## Data Availability

The data presented in this study are available on request from the corresponding author due to privacy reasons.

## References

[1] Ota M.O.C., Badur S., Romano-Mazzotti L., Friedland L.R. (2021). Impact of COVID-19 pandemic on routine immunization. Ann. Med..

[2] DeSilva M.B., Haapala J., Vazquez-Benitez G., Daley M.F., Nordin J.D., Klein N.P., Henninger M.L., Williams J.T.B., Hambidge S.J., Jackson M.L. (2022). Association of the COVID-19 Pandemic with Routine Childhood Vaccination Rates and Proportion up to Date with Vaccinations Across 8 US Health Systems in the Vaccine Safety Datalink. JAMA Pediatr..

[3] Pingali C. (2023). Vaccination coverage among adolescents aged 13–17 years—National Immunization Survey—Teen, United States, 2022. MMWR Morb. Mortal. Wkly. Rep..

[4] Daniels V., Saxena K., Roberts C., Kothari S., Corman S., Yao L., Niccolai L. (2021). Impact of reduced human papillomavirus vaccination coverage rates due to COVID-19 in the United States: A model based analysis. Vaccine.

[5] Robinson R., Nguyen E., Wright M., Holmes J., Oliphant C., Cleveland K., Nies M.A. (2022). Factors contributing to vaccine hesitancy and reduced vaccine confidence in rural underserved populations. Humanit. Soc. Sci. Commun..

[6] Ekezie W., Connor A., Gibson E., Khunti K., Kamal A. (2023). A systematic review of behaviour change techniques within interventions to increase vaccine uptake among ethnic minority populations. Vaccines.

[7] Gray A., Fisher C.B. (2022). Determinants of COVID-19 vaccine uptake in adolescents 12–17 years old: Examining pediatric vaccine hesitancy among racially diverse parents in the United States. Front. Public Health.

[8] Glatman-Freedman A., Nichols K. (2012). The effect of social determinants on immunization programs. Hum. Vaccines Immunother..

[9] Fisher K.A., Nguyen N., Fouayzi H., Singh S., Crawford S., Mazor K.M. (2023). Impact of a physician recommendation on COVID-19 vaccination intent among vaccine hesitant individuals. Patient Educ. Couns..

[10] Lu P., Hung M.-C., O’Halloran A.C., Ding H., Srivastav A., Williams W.W., Singleton J.A. (2019). Seasonal influenza vaccination coverage trends among adult populations, United States, 2010–2016. Am. J. Prev. Med..

[11] Puri N., Coomes E.A., Haghbayan H., Gunaratne K. (2020). Social media and vaccine hesitancy: New updates for the era of COVID-19 and globalized infectious diseases. Hum. Vaccines Immunother..

[12] Waring M.E., Blackman Carr L.T., Heersping G.E. (2023). Social media use among parents and women of childbearing age in the US. Prev. Chronic Dis..

[13] Garcia S., Shin M., Sloan K., Dang E., Garcia C.O., Baezconde-Garbanati L., Palinkas L.A., Crabtree B.F., Tsui J. (2023). Disruptions to and innovations in HPV vaccination strategies within safety-net healthcare settings resulting from the COVID-19 pandemic. Healthcare.

[14] Matoff-Stepp S., Applebaum B., Pooler J., Kavanagh E. (2014). Women as health care decision-makers: Implications for health care coverage in the United States. J. Health Care Poor Underserved.

[15] Gottfried J. Americans’ Social Media Use. Pew Research Center. Published 31 January 2024. https://www.pewresearch.org/internet/2024/01/31/americans-social-media-use/.

[16] R Core Team R: A Language and Environment for Statistical Computing. Published online 2022. https://www.R-project.org.

[17] Vasudevan L., Ostermann J., Wang Y., Harrison S.E., Yelverton V., Fish L.J., Williams C., Walter E.B. (2022). Association of caregiver attitudes with adolescent HPV vaccination in 13 southern US states. Vaccine X.

[18] Lasher E., Fulkerson G., Seale E., Thomas A., Gadomski A. (2022). COVID-19 vaccine hesitancy and political ideation among college students in Central New York: The influence of differential media choice. Prev. Med. Rep..

[19] Head K.J., Kasting M.L., Sturm L.A., Hartsock J.A., Zimet G.D. (2020). A national survey assessing SARS-CoV-2 vaccination intentions: Implications for future public health communication efforts. Sci. Commun..

[20] Choi J., Lieff S.A., Meltzer G.Y., Grivel M.M., Chang V.W., Yang L.H., Des Jarlais D.C. (2022). Anti-vaccine attitudes among adults in the U.S. during the COVID-19 pandemic after vaccine rollout. Vaccines.

[21] Roberts H.A., Clark D.A., Kalina C., Sherman C., Brislin S., Heitzeg M.M., Hicks B.M., Gesser-Edelsburg A. (2022). To vax or not to vax: Predictors of anti-vax attitudes and COVID-19 vaccine hesitancy prior to widespread vaccine availability. PLoS ONE.

[22] Wilson S.L., Wiysonge C. (2020). Social media and vaccine hesitancy. BMJ Glob. Health.

[23] Romer D., Jamieson K.H. (2020). Conspiracy theories as barriers to controlling the spread of COVID-19 in the U.S. Soc. Sci. Med..

[24] Reno C., Maietti E., Di Valerio Z., Montalti M., Fantini M.P., Gori D. (2021). Vaccine hesitancy towards COVID-19 vaccination: Investigating the role of information sources through a mediation analysis. Infect. Dis. Rep..

[25] Middleman A.B., Klein J., Quinn J. (2022). Vaccine hesitancy in the time of COVID-19: Attitudes and intentions of teens and parents regarding the COVID-19 vaccine. Vaccines.

[26] Ortiz R.R., Smith A., Coyne-Beasley T. (2019). A systematic literature review to examine the potential for social media to impact HPV vaccine uptake and awareness, knowledge, and attitudes about HPV and HPV vaccination. Hum. Vaccines Immunother..

[27] Grosso F.M., Baldassarre M.E., Grosso R., Di Mauro F., Greco C., Greco S., Laforgia N., Di Mauro A. (2023). Do social media interventions increase vaccine uptake?. Front. Public Health.

[28] Buller D.B., Pagoto S., Henry K., Berteletti J., Walkosz B.J., Bibeau J., Baker K., Hillhouse J., Arroyo K.M. (2021). Human papillomavirus vaccination and social media: Results in a trial with mothers of daughters aged 14–17. Front. Digit. Health.

[29] Gilkey M.B., McRee A.-L. (2016). Provider communication about HPV vaccination: A systematic review. Hum. Vaccines Immunother..

[30] Gilkey M.B., Calo W.A., Moss J.L., Shah P.D., Marciniak M.W., Brewer N.T. (2016). Provider communication and HPV vaccination: The impact of recommendation quality. Vaccine.

[31] Reno J.E., O’Leary S., Garrett K., Pyrzanowski J., Lockhart S., Campagna E., Barnard J., Dempsey A.F. (2018). Improving provider communication about HPV vaccines for vaccine-hesitant parents through the use of motivational interviewing. J. Health Commun..

[32] Healy C.M., Pickering L.K. (2011). How to communicate with vaccine-hesitant parents. Pediatrics.

[33] Constable C., Ferguson K., Nicholson J., Quinn G.P. (2022). Clinician communication strategies associated with increased uptake of the human papillomavirus (HPV) vaccine: A systematic review. CA Cancer J Clin..

[34] Fontenot H.B., Quist K.M., Glauberman G., Michel A., Zimet G. (2024). Impact of the COVID-19 pandemic on social media utilization, influences related to parental vaccine decision making, and opinions on trustworthy social media vaccination campaigns: A qualitative analysis. Hum. Vaccines Immunother..

